# Bacteriophage Therapy of Bacterial Infections: The Rediscovered Frontier

**DOI:** 10.3390/ph14010034

**Published:** 2021-01-05

**Authors:** Nejat Düzgüneş, Melike Sessevmez, Metin Yildirim

**Affiliations:** 1 Department of Biomedical Sciences, Arthur A. Dugoni School of Dentistry, University of the Pacific, San Francisco, CA 94103, USA; 2 Department of Pharmaceutical Technology, Faculty of Pharmacy, Istanbul University, Istanbul 34116, Turkey; melikesessevmez@gmail.com; 3 Department of Pharmacy Services, Vocational School of Health Services, Tarsus University, Mersin 33400, Turkey; metinyildirim4@gmail.com

**Keywords:** lytic infection, antibiotic-resistance, *Mycobacterium tuberculosis*, *Acinetobacter baumannii*, *Pseudomonas aeruginosa*, phage production, magistral phage, pulmonary delivery, oral administration, topical delivery

## Abstract

Antibiotic-resistant infections present a serious health concern worldwide. It is estimated that there are 2.8 million antibiotic-resistant infections and 35,000 deaths in the United States every year. Such microorganisms include *Acinetobacter*, Enterobacterioceae, *Pseudomonas*, *Staphylococcus* and *Mycobacterium*. Alternative treatment methods are, thus, necessary to treat such infections. Bacteriophages are viruses of bacteria. In a lytic infection, the newly formed phage particles lyse the bacterium and continue to infect other bacteria. In the early 20th century, d’Herelle, Bruynoghe and Maisin used bacterium-specific phages to treat bacterial infections. Bacteriophages are being identified, purified and developed as pharmaceutically acceptable macromolecular “drugs,” undergoing strict quality control. Phages can be applied topically or delivered by inhalation, orally or parenterally. Some of the major drug-resistant infections that are potential targets of pharmaceutically prepared phages are *Pseudomonas aeruginosa*, *Mycobacterium tuberculosis* and *Acinetobacter baumannii*.

## 1. Introduction: Bacteriophage Treatment of a Serious Infection

A 68-year-old man with diabetes developed necrotizing pancreatitis that was complicated by a pancreatic pseudocyst infected with a multi-drug-resistant strain of *Acinetobacter baumannii* [1]. *A. baumannii* is a Gram-negative nosocomial pathogen involved in bacteremia, meningitis and pulmonary infections with a high mortality rate. It is one of the “ESKAPE” microorganisms that are grouped together because of the common occurrence of multi-drug-resistance in the group. These microorganisms include *Enterococcus faecium*, *Staphylococcus aureus*, *Klebsiella pneumoniae*, *Acinetobacter baumannii*, *Pseudomonas aeruginosa* and *Enterobacter* species. The condition of the patient was deteriorating rapidly despite antibiotic treatment, to which he was obviously not responding. Bacteriophage therapy was initiated as part of an emergency investigational new drug protocol.

**Figure 1:**
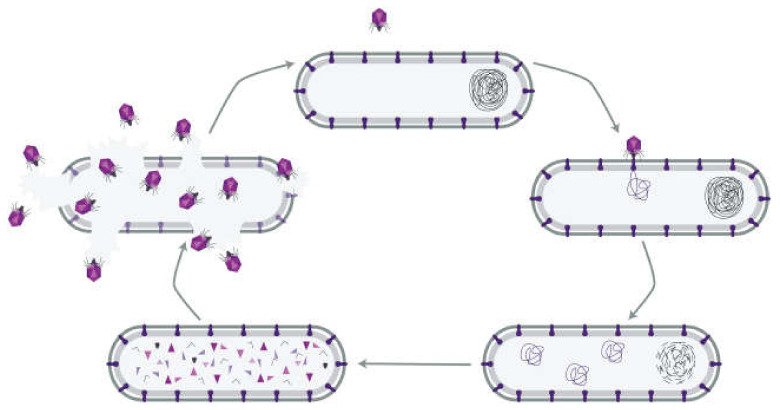
The lytic infection cycle of a bacteriophage. A phage particle attaches to a receptor on the surface of a host bacterium and delivers its genome into the cytoplasm. Phage proteins and replicate genomes are synthesized and self-assemble into new phage particles that eventually lyse the bacterium. The phages then infect other bacteria with the particular receptor (reproduced with permission from Kortright et al., 2019 [4]).

Bacteriophages are viruses of bacteria. Phages can cause either lytic or lysogenic infections in bacteria after attaching to a receptor or receptors on the bacterial surface and delivering their genome into the bacteria. In a lytic infection, the phage replicates and the new phage particles lyse the bacterium and continue to infect other bacteria (Figure 1). In a lysogenic infection, a DNA phage inserts its genetic material into the bacterial chromosome, and the genome is passed on to daughter cells as the bacterium divides. The integrated DNA may be activated by changes in environmental conditions to excise itself from the chromosome, producing phage particles that become lytic [2,3,4].

A large number of phage types that could specifically lyse *A. baumannii* were tested against three strains of the bacterium obtained previously from the patient. The phages had been collected by the Biological Defense Research Directorate of the Naval Medical Research Center. Most of the phages, however, were not lytic towards the first clinical bacterial sample obtained from the patient. Six phage types inhibited bacterial growth for 20 h. Four of these phages were pooled and, when tested against the bacterial isolate from the patient, had superior activity compared to each of the phages by themselves. The patient showed clear clinical improvement within 2 days of the administration of the phage cocktail containing a total of 5 × 10^9^ particles. Initially, the phage mixture was delivered through the percutaneous catheters draining the pseudocyst cavity, the gallbladder and the intra-abdominal cavity and repeated every 6 to 12 h. Thirty-six hours after the initiation of treatment, the phage cocktail was given intravenously [1]. This treatment reversed the patient’s clinical decline, cleared the *A. baumannii* infection and returned the patient to health.

The British bacteriologist Ernest Hankin (1896) noted the anti-*Vibrio cholerae* activity of water from two rivers in India and suggested that a substance that could pass through porcelain filters caused this observation and possibly limited cholera epidemics.

The Russian bacteriologist Gamaleya reported a similar phenomenon with *Bacillus subtilis* [5]. The English bacteriologist Frederick Twort hypothesized in 1915 that the antibacterial effect could be mediated by a virus [6]. The French-Canadian microbiologist Felix d’Herelle, at the Pasteur Institute in Paris, identified non-bacterial microorganisms from the stools of patients suffering from severe hemorrhagic dysentery. These microorganisms formed plaques in cultures of *Shigella* isolated from the patients [7]. In 1919, d’Herelle used a phage preparation to treat a boy with dysentery who recovered within a day and then three additional patients who started to recover within a day [8,9]. The first report of phage therapy was published in 1921 by Richard Bruynoghe and Joseph Maisin [10]. They injected bacteriophages into and around surgically opened staphylococcal skin lesions, which regressed within 1–2 days.

## 2. Phages as Pharmaceuticals

### 2.1. Phage Isolation and Enrichment

The key processes in phage therapy protocols are phage selection and isolation. The wrong choices can have fatal consequences [11]. Generally, two methods are used when choosing the appropriate phage for therapy: (1) A phage cocktail, such as Pyophage and Intestiphage. These preparations have a broader spectrum of activity than a single phage component and do not allow resistance to develop within a short time. (2) A pathogen-specific phage. Bacteria are isolated from the infection and tested for susceptibility to particular phages isolated previously [11].

**Figure 2:**
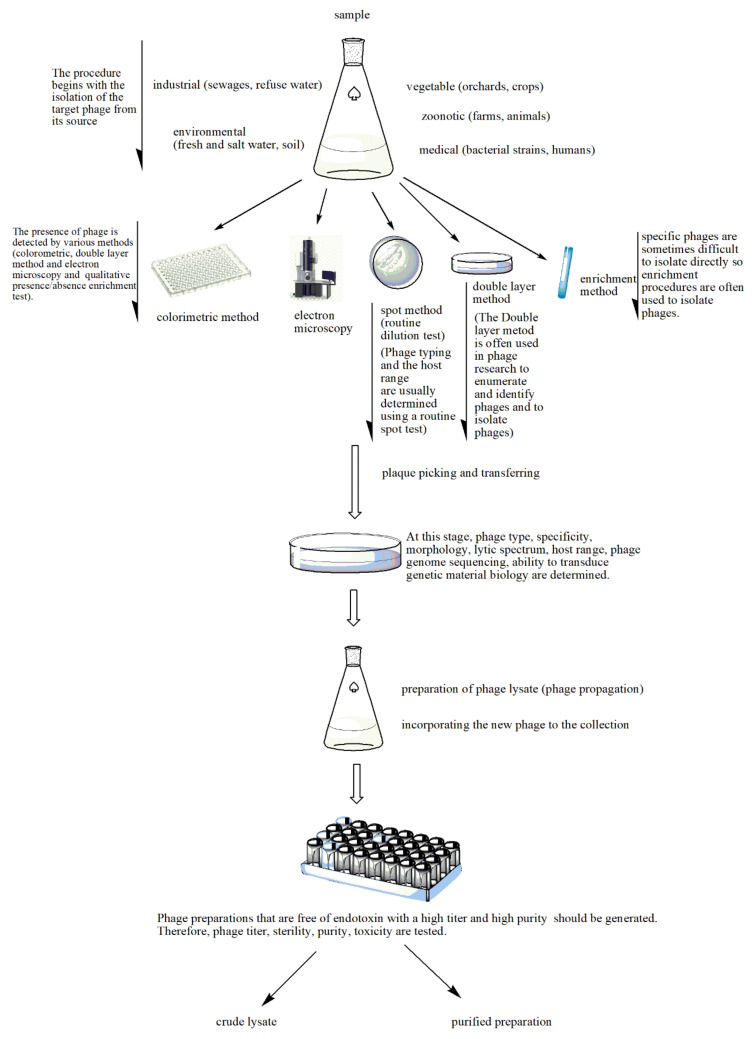
Stages of phage preparation. The environment (e.g., wastewater, farms and soil) is a source for all types of phages. The presence of phages in the tested sample is determined by different methods such as the double layer agar method, spot assays, the colorimetric method, the enrichment method or electron microscopy. The plaques that indicate lytic activity are picked up and transferred for the determination of phage type, specificity, etc. A phage lysate is prepared. At this stage, multiple procedures are performed to check for sterility (microbial contamination), toxicity (bacterial endotoxin or lipopolysaccharide (LPS) quantification), bacterial DNA contamination and phage titer. The purified phage preparation is stored.

Samples for phage isolation are taken from environments where the bacterial host can often be found, including soil, plant residues, fecal matter, wastewater and sewage (Figure 2). Phages against *Shigella dysenteriae* 2308 were isolated from the New York City sewage by Dubos et al. [12]. B_VpS_BA3 and vB_VpS_CA8 phages against *Vibrio parahaemolyticus* were isolated from sewage collected in China [13]. The vB_KpnP_IME337 phage against carbapenem-resistant *Klebsiella pneumoniae* was isolated from hospital sewage in China [14]. Li et al. [15] isolated 54 novel phages against the same organism from medical and domestic sewage wastewater. The newly isolated phage P545 had a relatively wide host range and strong antibacterial activity.

Although there are differences in phage isolation, the basic principle of the methods is the same as that developed by d’Herelle, and they are generally characterized as enrichment procedures [16]. First, the presence of phages is detected in the collected sample. Selection of the bacterial host is vital for the isolation of a phage in a recently acquired sample from the environment. Solid samples are mixed with sterile broth or buffer and then subjected to centrifugation and filtration [17]. Bacteria of interest are incubated overnight with the environmental sample. The bacteria that have survived the attack of the lytic phages are removed from the mixture by centrifugation or filtration, or both. The presence of phages in the filtrate is then assessed by plaque assay or by qPCR. The isolated phages have to be analyzed for their virulence, i.e., their ability to lyse target bacteria and the range of bacterial types they are able to infect. In an alternate method, samples from the environment are plated directly onto a lawn of particular bacteria and the presence of plaques resulting from bacterial lysis is detected. The latter method has been used to discover phages that lyse *Escherichia coli* and various bacteria from dental plaque and the oral cavity [17,18].

In the procedure described in detail by Luong et al. [19], the target bacterial strain is isolated and incubated with the phages. Then, after several agar plaque isolations, a single plaque is cultivated overnight. The isolated phage genome is sequenced to screen and identify lysogenic and deleterious genes. Phages are grown at liter scale, and the lysate is purified to eliminate any bacteria and cellular debris by pressure-driven filtration through filters of 0.8-, 0.45- and 0.22-µm pore size, followed by cross-flow ultra-filtration to eliminate debris smaller than 100 kD. This process eliminates endotoxin, exotoxins, peptidoglycan, nucleic acids and flagella. The phage particles are purified by CsCl density gradient centrifugation and dialysis to eliminate the CsCl. Any residual endotoxin molecules are removed by lipopolysaccharide-affinity chromatography. The last step ensures that the phage preparations do not cause inflammation or endotoxic shock when administered to patients [19].

Phages are expected to be found where the host bacteria reside. For example, phages that infect intestinal bacteria can be isolated from fecal material, and phages against epidermal bacteria such as *Staphylococcus aureus* are most likely isolated from skin samples or wound exudates. Identifying a phage against a particular bacterium is not straightforward, however. Whereas phages that lyse antibiotic-resistant *Klebsiella pneumoniae* and *Pseudomonas aeruginosa* were readily isolated from sewage samples, phages against antibiotic-resistant *Acinetobacter baumannii* were not found as frequently [20]. Furthermore, phages against methicillin-resistant *Staphylococcus aureus* were identified only rarely.

The choice of a host for phage isolation may also depend on the ease of culturing the bacteria, as in the use of *Mycobacterium smegmatis* to isolate phages that will infect other *Mycobacterium* species. *M. smegmatis* grows much faster than *M. tuberculosis* and thus can produce a lawn on the appropriate agar surface for testing phage activity [17,20,21]. The isolated phages would then be tested further on the specific target *Mycobacterium* species.

Swanstrom et al. [22] investigated the variables contributing to the generation of high-titer phage stocks, using the agar layer method and coli phage T4r. The numbers of virus particles and bacteria per plate, the incubation period, the amount of soft agar in the agar layer as well as the broth volume used for virus extraction from the agar were found to be significant factors. When these factors were optimized, stock concentrations in the range of 10^11^–10^12^ infectious particles/mL could be obtained [22].

Echeverría-Vega et al. [23] used a straightforward protocol for the isolation of bacteriophages from coastal organisms. They also validated the protocol for the isolation of lytic bacteriophages for the fish pathogen bacterium *Vibrio ordalii*. This method has particular utility for the recovery of bacteriophages for use as natural antimicrobial agents in aquacultures. In the enrichment method, samples are added to the host produced in a suitable medium, incubated and then centrifuged. The suspension containing the phage is filtered and applied at different concentrations onto an agar medium with target bacteria. The formed plaques are then counted. Thanks to the enrichment method, phages at low concentration can reach the desired level in culture. Enrichment is an advantageous method in cases where the amount of phages is low [24]. Numerous lytic phages were isolated against *Caulobacter* and *Asticcacaulis* bacteria using the enrichment method [25]. Methods such as spot testing, plaque testing, culture lysis and the calorimetric method are used in the detection of newly isolated bacteriophages (Figure 2) [26,27,28,29,30,31]. In the spot assay [26], bacteria are grown in Luria-Bertani broth, and after they are in the early log phase, they are mixed with soft agar and poured onto a Petri plate with previously poured agar. A phage filtrate is then placed on the soft agar and the plates are incubated overnight at 37 °C, after which bacterial lysis zones are counted [28]. In the double layer agar method, a bacterial culture in the log phase is mixed with a purified phage preparation and incubated briefly to allow for phage adsorption. This mixture is combined with soft agar and poured onto a previously solidified agar layer to form a homogeneous layer. After incubation at 37 °C for 24 h, plaque formation is observed, indicating phage activity. The plaques are resuspended in Mg (SM) buffer [28].

### 2.2. Phage Production

Bacteriophages need a host cell to reproduce. Understanding the interactions between host bacteria and bacteriophages is a crucial step in estimating the risks in production, including possible mutations in either microorganism [32]. The production process may also be affected by the nutrient composition, oxygenation, temperature and pH [33].

The substrate and temperature chosen for phage infection and bacterial growth are important factors. Fermentation is an important stage for host bacteria to multiply and produce bacteriophages. The sterilization step is performed to destroy undesired microorganisms. The bioreactor can be sterilized with heat, medium or a combination of these. During the fermentation process, the injected air is filtered through an in-line membrane. The air released after fermentation is filtered after it condenses [34].

Phages are grown basically in shaker flasks or stirred tank bioreactors. The latter are used to carry out industrial-scale production of bacteriophages, which has been divided into three different systems: batch, semi-continuous and continuous [35]. Each system has brought about its distinct benefits and drawbacks, discussed earlier by Merabishvili et al. [36]. Mancuso et al. [37] developed a production process that makes it possible to obtain high titers of *E. coli* T3 phages at high concentrations (10^11^ PFU mL^−1^) using two continuous stirred tank bioreactors. The first bioreactor is just for propagation of the host bacteria at a steady-state growth rate by using controllable dilution rates and growth-limiting substrate (glucose). The second bioreactor is used for bacteriophage production and is fed from the host bacteria of the first bioreactor. Besides achieving high phage productivity of bacteriophages via the production process, the mutation risk of the host bacteria potentially caused by bacteriophages is suppressed.

### 2.3. Phage Purification and Quality Control

For the pharmaceutical application of phages, it is necessary to first carry out the purification process. The bacteriophage of interest is separated from host bacteria cells and debris by centrifugation, microfiltration or by using these methods together. The potential presence of any toxins in the preparations would be detrimental to the final pharmaceutical product. A Chamberland filter of 0.1–1 µm was used for bacteriophage preparations to be used in human trials [38]. It was recently clarified with a 0.2-μm filter pore size. Purification procedures of phages should follow the Critical Quality Attributes (CQA) specification [34]. The process of removing endotoxins from phages is complex because lipopolysaccharide forms micelles that have approximately the same size as phages. Therefore, extra purification methods such as ion exchange, affinity chromatography and solvent extraction are needed for lysates of phage-infected Gram-negative bacteria [33].

Endotoxin. In bacteriophage products, endotoxin measurement is critical. Gel clot, turbidimetric and chromatic methods are used for endotoxin determination in bacteriophage products. The *Limulus* amoebocyte lysate (LAL) assay is the most commonly used method [39].

Transmission electron microscopy (TEM). The specific morphology of phages in a final product can be viewed by transmission electron microscopy. Merabishvili et al. [40] used TEM for confirmation of the presence of the expected virion morphologic particles as well as their specific interaction with the target bacteria.

Titer. The process of determining phage concentration by dilution and plating with susceptible cells is called titering or the plaque assay. A bacteriophage capable of productively infecting a cell is named a plaque-forming unit (PFU/mL) [41].

pH. In a therapeutic formulation, the pH value is very important. According to the European Pharmacopoeia, the pH should be in the range 6.0–8.0 [42].

Nucleic Acid Contaminants. Because phages break down bacterial DNA, the presence and concentration of nucleic acid residues in final products should be determined. qPCR can be used for this purpose [33].

### 2.4. Phage Stability and Storage Conditions

Once solutions of phages are prepared, the biological properties of the phages have to be preserved during storage. Freeze-drying, spray-drying or encapsulation methods can be used to increase phage stability, as well as adding stabilizing additives to their solutions [43,44,45,46]. The quality, safety and storage conditions of phages to be prepared for use in treatment should be validated [47]. González-Menéndez et al. [48] investigated different preservation techniques for the storage of *Staphylococcus* phages (phiIPLA88, phiIPLA35, phiIPLA-RODI and phiIPLA-C1C). They evaluated the stability of phages at different temperatures (−20, −80 and −196 °C) and time periods (1, 6, 12 and 24 months). They also investigated various stabilization enhancing agents, including disaccharides, glycerol, sorbitol and skim milk. They showed that at −80 and −196 °C, all phages showed good viability after 24 months, regardless of the stabilizer [48].

### 2.5. Therapeutic Phages

Hyman et al. [17] proposed the following characteristics of phages to be used for therapeutic purposes: (a) The phage should be virulent and be able to cause complete cytotoxicity to the target bacterium. (b) It should be exclusively lytic and should not become temperate (i.e., lysogenic). (c) The phage should have the potential to transduce the host bacteria. (d) It should have the desired host range. (e) It should be screened for toxin genes that can affect the patient. *Myoviridae, Siphoviridae and Podoviridae* families are used commonly for phage therapy [49,50]. There are approximately 800 phages against pathogens such as *Escherichia*, *Morganella*, *Klebsiella*, *Enterococcus*, *Pseudomonas*, *Staphylococcus* and *Salmonella* [31].

### 2.6. Magistral Phage

A “magistral preparation” or a “compounded prescription drug product”, in Europe and the U.S., respectively, is defined as “any medicinal product prepared in a pharmacy in accordance with a medical prescription for an individual patient” [33,34,48,51,52]. Such preparations for a particular patient are mixed by a pharmacist from their individual ingredients based on a prescription from a physician. The magistral formula is a practical way for a medical doctor to personalize patient treatments to specific needs and to make medications available that do not exist commercially. Some medicines, including natural hormone combinations, are made as magistral preparations. It is expected that magistral preparations will become more readily available as novel medicines are developed to treat rare conditions.

A magistral phage preparation is prepared from a phage bank, which is a repository of well-characterized microorganisms. A phage as an active pharmaceutical ingredient (API) is produced using a suitable bacterial host. An approved laboratory then carries out External Quality Assessments to test the API’s properties and quality. Active phage APIs are evaluated for activity against the target. Finally, phage APIs are mixed with a suitable carrier system. There are currently no guidelines on the preparation, formulation and use of magistral phages [52].

### 2.7. Topical Administration of Phages

Several studies have shown that local and topical phage applications are successful. In the treatment of infections caused by Staphylococci, *Klebsiella*, *Pseudomonas*, *Proteus* and *Escherichia* such as conjunctivitis, otitis, gingivitis, furunculosis, decubitis ulcer, open wound infection, burns, osthitis (caused by fractures) and chronic suppurative fistulae, phage cocktails have been applied locally [53,54,55,56]. A commercial product called PhagoBioDerm, which targets *P. aeruginosa*, *S. aureus* and *Streptococcus* spp. and contains phages as well as cipro-floxacin, can be applied directly over infected wounds. Goode et al. [57] eliminated *Salmonella* contamination on chicken skin by using a lytic bacteriophage. Vieira et al. [58] performed phage therapy against multidrug-resistant *P. aeruginosa* that had caused skin infections. Thanks to phage therapy, the amount of *P. aeruginosa* 709 present in human skin decreased by four orders of magnitude.

### 2.8. Pulmonary Phage Delivery

The first studies of inhaled phage therapy were carried out in the early 1960s. Such treatments at a more advanced level were performed in Russia, Poland and Georgia. Although there have been many successful trials, some treatments have not had a positive outcome because of a lack of phage variety, quality control and technical knowledge [59]. Phages may be encapsulated in polymers, nanoparticles and liposomes for stability during storage, including storage as a freeze-dried preparation [60]. Liposome encapsulation was found to facilitate phage entry into macrophages. Treatment of experimental *K. pneumoniae*-induced lobar pneumonia was more effective with liposome-entrapped phages administered intraperitoneally as late as 3 days post-infection, whereas free phages provided a therapeutic effect only if they were administered at 1 day after infection [61]. Systemic side effects were reduced by the use of liposomal phage.

Liquid formulations using intranasal instillation and nebulization in phage studies against respiratory infections on animal models are quite popular. Liquid phage formulations are stable, easily aerosolizable and easy to formulate compared to other carrier systems [59]. Carrigy et al. [62] tested pre-exposure prophylactic aerosol delivery of the anti-tuberculosis bacteriophage D29 as an option for protection against *Mycobacterium tuberculosis* infection and proposed that mycobacteriophage aerosols at sufficient doses may be protective against *M. tuberculosis* infection. The same group studied the titer reduction and phage delivery rate of three inhalation devices (Vibrating Mesh Nebulizer, Jet Nebulizer and Soft Mist Inhaler) with the mycobacteriophage D29 and showed that this method of administration is suitable for phage delivery to lung tissue [63]. Golshahi et al. [64] determined that the inhaled formulation of bacteriophages gives successful results in the treatment of cystic fibrosis pulmonary infections.

### 2.9. Parenteral Phage Application

Phages are rapidly eliminated by the immune system when administered intravenously. Lin et al. [65] investigated the intravenous administration of the anti-pseudomonal phage øPEV20 in *P. aeruginosa*-infected rats and demonstrated dose-dependent pharmacodynamics. Intravenous administration of øPEV20 at a dose of >10^4^ PFU/mouse resulted in rapid bacterial killing and >8-log_10_ CFU/mL reduction in bacterial load compared with the initial inoculum and untreated controls at 2.5 h. However, treatment at a dose of <10^4^ øPEV20 PFU/mouse was ineffective against pan-drug-resistant *P. aeruginosa*. McVay et al. [66] injected a *P. aeruginosa* phage cocktail with three different administration methods (subcutaneous (s.c.), intramuscular (i.m.) and intraperitoneal (i.p.)) to *P. aeruginosa*-infected mice. Without treatment, the survival rate was 6%, and i.p. administration of phage resulted in the highest rate of survival (87%). According to the results of pharmacokinetic studies on phages, compared to other administration routes, phages reached the target at higher concentrations and faster when given via the i.p. route.

### 2.10. Oral Phage Therapy

Oral formulations of bacteriophages are generally used to target acute gastrointestinal infections. However, there are a number of factors for the treatment to be successful, including stability and effective phage dose at the site of infection. A significant decrease in phage titers occurs before the phages reach the site of infection. Phage viability and activity decrease as a result of gastric acidity and digestive enzymes such as pepsin and pancreatin. Thus, it is necessary to prepare new dosage forms. Vinner et al. [67] encapsulated enteric bacteriophage K1F against *E. coli* in a pH-responsive solid formulation and examined the viability of these bacteriophages at different pH values. They found that the microencapsulation process preserved phages for an extended period in the gastric acid environment. The encapsulated phages were active in killing *E. coli* co-incubated with human epithelial cells, which are normally stressed in the presence of the bacteria alone. There were no stability problems for the encapsulated phages that were refrigerated for 4 weeks. Stanford et al. [68] used polymer-encapsulated wV8, rV5, wV7 and wV11 phages, which are targeted to *E. coli*. Then, the phages were exposed to pH 3.2 for 20 min. The unencapsulated phages lost their activity while the encapsulated phages recovered 13.6% of their activity. Vinner et al. [69] prepared the encapsulated bacteriophage Felix O1, which is specific to *Salmonella*, by spray-drying, employing a commercially available pH-sensitive copolymer of methyl methacrylate and methacrylic acid. The inclusion of trehalose in the formulation protected the phages from the effects of spray-drying, maintaining the original phage titer. In a different approach, Colom et al. [70] encapsulated the phages UAB_Phi20, UAB_Phi78 and UAB_Ph87 individually in a complex mixture of lipids that produced a net positive charge on the ensuing liposomes. The encapsulation efficiencies were relatively high, in the range of 47–49%, which is most likely the result of phage binding to the net cationic lipid mixture. The encapsulated phages were more effective than plain phages in *S. enterica* ser. Typhimurium-infected chickens at only 8 days following treatment, with a 3.9 log_10_ reduction [70].

## 3. Mycobacteriophage Therapy of *Mycobacterium tuberculosis*

There are more than 170 *Mycobacterium* species that have great variety in terms of their pathogenicity in humans [71]. In addition to *M. tuberculosis*, *M. ulcerans* and *M. leprae* cause Buruli ulcer and leprosy, respectively [72]. *M. tuberculosis* is a well-known example of an intracellular bacterium that localizes inside phagosomes of macrophages of the host and causes tuberculosis (TB), which primarily affects the lungs [73]. Multi-drug-resistant (MDR) TB cases have emerged in the late 1980s and early 1990s. These strains are resistant to the first-line drugs against TB, rifampicin and isoniazid. In 2018, the World Health Organization (WHO) reported that 484,000 new TB patients failed to respond to rifampicin. Seventy-eight percent of these patients were infected with MDR-TB [74].

Alternative treatment approaches for MDR-TB have become crucial to managing the disease. One of these approaches is mycobacteriophage therapy. More than 70 years have passed since mycobacteriophages were isolated for the first time [75]. So far, 11,282 mycobacteriophages have been isolated [76].

**Figure 3:**
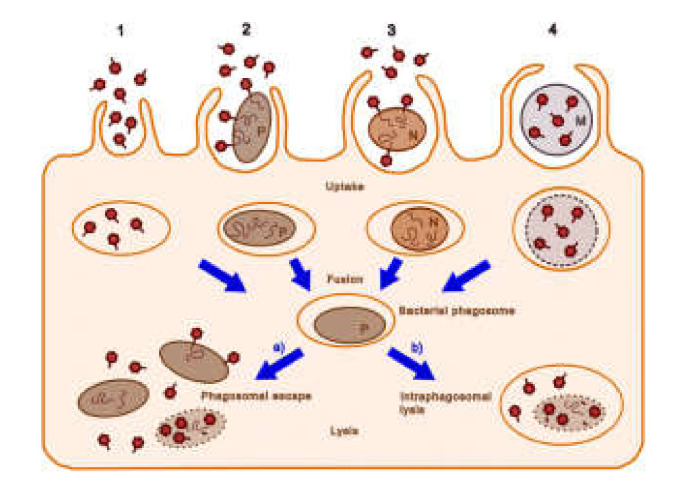
Possible cellular entry pathways of bacteriophages. The pathways 1–4 are described in the text. Dark red hexagons, bacteriophage; brown-filled ovals, bacteria (pathogenic bacteria, P; non-pathogenic bacteria, N); curly lines within bacteria, bacteriophage nucleic acids; orange ovals, endosomal vesicles; blue-gray circles and M, microcapsules; dotted lines, degrading bacterial membrane (reproduced with permission from Nieth et al., 2015 [77]).

Bacteriophages can enter macrophages by four main routes [77] (Figure 3): (1) Endocytotic uptake of the bacteriophage alone; (2) entry into the macrophage via pathogenic bacteria together with the bound phage; (3) uptake of the bacteriophage and non-pathogenic bacteria; (4) internalization of the bacteriophage that has been encapsulated by poly-mers or liposomes. Relatively non-pathogenic vectors, such as *M. smegmatis,* can be used to deliver phages to the same intracellular compartments where *M. tuberculosis* is found [78]. The lytic mycobacteriophage TM4 was delivered in this manner to *M. tuberculosis*-infected RAW264.7 macrophages and reduced the bacterial counts. By contrast, the phage alone was ineffective. The administration of the *M. smegmatis*-TM4 complex to *M. avium*-infected mice significantly decreased the bacterial counts in the spleen, whereas TM4 or *M. smegmatis* alone had no effect [79]. The authors suggested that phage resistance (which was observed in their study) could be overcome by the use of phage cocktails.

The mycobacteriophage D29 was used to treat *M. tuberculosis* H37Rv inside RAW 264.7 macrophages [80]. The phage, administered twice over a 24-h period, caused an eight-fold reduction in the CFUs, indicating that it was able to access the intracellular compartment occupied by the bacteria. The phage was encapsulated in (or associated with) liposomes comprising phosphatidylcholine, cholesterol and Tween-80 and which were sized by extrusion through membranes of 400-nm diameter. This formulation applied to infected macrophages resulted in a two-fold improvement of the antimycobacterial effect over that of the free phage. In an in vitro model of tuberculous granuloma developed from peripheral blood mononuclear cells of patients with TB, liposomal phage was about nine-fold more effective than free D29 [81].

Aerosolized bacteriophage D29 was used to investigate the possibility of protecting mice against *M. tuberculosis* infection [62]. This treatment significantly decreased the *M. tuberculosis* counts in the lungs 1 day and 3 weeks after challenge. The authors suggested that aerosolized mycobacteriophages may be useful in conferring additional protection to healthcare workers who may be at risk of exposure to tuberculosis. D29 was also employed in a murine footpad model in treating Buruli ulcer, which is caused by *Mycobacterium ulcerans* [82]. In infected patients, the bacterium causes necrosis of the skin, subcutaneous tissue as well as bone. If the disease reaches advanced stages, surgical resection of the skin may be necessary. The subcutaneous injection of D29 resulted in a decrease in pathology and mycobacterial counts. It also caused increased production of cytokines, including IFN-γ, in the footpads and draining lymph nodes. Endolysins are bacteriophage-encoded peptidoglycan-disrupting enzymes synthesized at the last stage of the phage life cycle in the infected bacteria [83]. One endolysin, lysine B, was found to lyse *M. ulcerans* infecting the footpad of experimental mice [84].

Developing mycobacteriophages into efficient therapeutic pharmaceuticals has focused on improving their uptake into macrophages and co-localization with the intracellular mycobacteria. In this process, however, it is essential to maintain the stability of the formulation and the vitality of the mycobacteriophages. In the next step, well-established in vitro and in vivo studies of effective and stable mycobacteriophage formulations are expected to translate into clinical studies with successful outcomes.

## 4. Bacteriophage Therapy of *Pseudomonas aeruginosa*

*P. aeruginosa* are Gram-negative aerobic bacteria classified as Gammaproteobacteria that can cause severe necrotizing bronchopneumonia, burn wound infections, urinary tract infections, otitis externa, eye infections and bacteremia [85].

In a murine model of sepsis caused by *P. aeruginosa* via the gut, the lytic phage KPP10 administered orally increased the survival rate from 0% in the controls to 67% [86]. The number of viable bacteria in the liver, spleen and blood were reduced in the phage-treated group, as were the levels of inflammatory cytokines in the liver and blood. Imipenem-resistant *P. aeruginosa* delivered i.p. resulted in bacteremia and killed 100% of experimental mice within 24 h [87]; the i.p. administration of the phage ØA392 within 1 h of infection was able to rescue all the animals. The phages were found in blood within 2 h. However, delivery of the phage at 3 h post-infection resulted in only 50% survival. In a murine burn-wound model, fatal infection by *P. aeruginosa* could be reduced to 87% survival when a three-phage cocktail was given i.p. [67]. The phages rapidly distributed to the blood, liver and spleen. In a similar study, i.p. delivery of multi-drug-resistant *P. aeruginosa* caused fatal bacteremia in mice within 2 d [88]. A phage strain that had lytic activity against numerous multi-drug-resistant *P. aeruginosa* given 45 min after bacterial infection resulted in 100% survival. Fifty percent of the animals could be saved even when the therapy was applied at a point where the animals were sick. The therapeutic effect of the phage was also shown not to be the result of a non-specific immune response.

When mice with acute lung infection with intranasally administered bioluminescent *P. aeruginosa*, which resulted in the death of all the animals within 2 days, were treated with bacteriophage PAK-P1-to-bacterium ratios of 1:1 and 10:1 via the same route, they survived until the end of the 12-day experiment [89]. Bacteriophage treatment also prevented lung infection when administered 24 h before inoculation of bacteria. Two phages were isolated from wastewater, the myovirus φNH-4 and the podovirus φMR299-2, and used to treat *P. aeruginosa* infection in murine lungs [90]. The pathogen was reduced by three to four orders of magnitude in 6 h. A mixture of the two phages could kill biofilms of mucoid and nonmucoid strains of *P. aeruginosa* on CFBE41o-cystic fibrosis bronchial epithelial cells, and the phages were shown to multiply over 24 h.

Phage GNCP treatment of multi-drug-resistant *P. aeruginosa* infection in diabetic and non-diabetic mice, which caused fatal bacteremia within 2 d, at a 10:1 ratio of phage:bacteria resulted in protection of 90% of diabetic animals and 100% of non-diabetic animals [91]. Bacteriophages were also effective in reducing inflammation in a murine acute infection model of *P. aeruginosa* [92]. The titer of phage PEV31 delivered intratracheally to mice without bacterial infection decreased with a t_1/2_ of about 8 h. In mice infected with *P. aeruginosa*, the phage titer increased by about two orders of magnitude in 16 h, and bacterial growth was suppressed, whereas it increased exponentially in the untreated animals [93].

## 5. Clinical Cases Treated with Bacteriophages

Lung transplant recipients with life-threatening multi-drug-resistant *P. aeruginosa* or *Burkholderia dolosa* infections were treated with lytic bacteriophages targeting the bacterial strains, together with antibiotics [94]. Two patients with *P. aeruginosa* infection responded to the treatment and could leave the hospital. The patient with recurrent *B. dolosa* infection did not respond to bacteriophage therapy. The safety and feasibility of phage treatment of patients with various infections at a single center in the U.S. was established, although two of the 10 patients described did not respond to therapy [95].

*P. aeruginosa* can infect prosthetic vascular grafts that often do not respond to antibiotic therapy [96]. The bacteriophage OMKO1, together with ceftazidime, was used to treat infection of an aortic graft, which was resolved and did not recur.

Bacteriophage therapy was applied to chronic non-healing wounds that were infected with *E. coli, S. aureus* and *P. aeruginosa* and that did not respond to antibiotic therapy [97]. The application of a cocktail of bacteriophages over the wounds every other day resulted in the resolution of the infection after 3–5 doses. The wounds healed completely in seven out of 20 patients and formed healthy granulation tissue and margins in the other patients.

In a trial that included 48 patients with non-healing wounds, a single phage against a particular bacterial infection or multiple phages targeting multiple bacteria were applied every other day for 5 to 7 days [98]. The cure rate was 81%, with diabetic patients having a lower rate (74%).

A 65-year-old woman with a post-operative left-eye corneal abscess and interstitial keratitis was treated for many years with various antibiotics but remained positive for vancomycin-intermediate sensitivity *S. aureus* in the nasal cavity, skin and eye [99]. She then underwent topical and intravenous phage therapy with the bacteriophage SATA-8505. This phage strain is active against the methicillin-resistant *S. aureus* strain USA300 and has been patented. Ocular and nasal cultures from the patient 3 and 6 months after therapy showed no infection.

## 6. Conclusions

The importance of phage therapy for bacterial infections has been recognized by both academic institutions and the pharmaceutical industry. The Center for Phage Applications and Therapeutics at the University of California San Diego, the Center for Phage Technology at Texas A&M University at College Station and the Pittsburgh Bacteriophage Institute at the University of Pittsburgh are examples of academic institutions. Companies focusing on phage therapy include the Eliava Institute and affiliated companies in Tblisi, InnoPhage in Porto, Adaptive Phage Therapeutics in Gaithersburg, Intralytics in Columbia, Maryland, and Armata Pharmaceuticals in Marina Del Ray. Thus, it appears that phage therapy will be widely available, next to newly developed antibiotics, to teat multi-drug-resistant infections.

Although small-scale studies have demonstrated the potential of phage therapy for bacterial infections, especially in cases of severe antibiotic resistance, the widespread applicability of this therapy has not been shown in clinical trials [100]. In a clinical trial involving patients with urinary tract infections, phages administered directly into the bladder were no more effective than placebo or antibiotics [101]. Burn-wound infections with *P. aeruginosa* were treated with either the phage PP1131 or 1% sulfadiazine silver emulsion cream, the standard of care, in a multi-center clinical trial. Phage treatment at the relatively low dose of 10^6^ plaque-forming units per mL was not as effective as the standard of care [102].

To supplement phage therapy, it may be possible to utilize antibiotics and phages simultaneously in some circumstances. In an in vitro study of *P. aeruginosa* and *S. aureus* biofilms, either alone or in combination, the phage EPA1 that infects *P. aeruginosa* and different antibiotics, the simultaneous application of the two agents drastically increased the cytotoxicity against the bacteria [103]. The addition of gentamicin or ciprofloxacin after a 6-h treatment with the phage appeared to eradicate the bacterial biofilms, with higher gentamicin concentrations being necessary for treating combined biofilms.

Clinical trials of bacteriophage therapy of bacterial infections are still at an early stage. Optimal conditions of phage use, including their concentration, the time and sequence of administration and their combination with the appropriate antibiotics, are likely to establish the effectiveness and reliability of this medicine. Even until such standards are established, their ability to save the patient described in the Introduction is a most welcome addition to the practice of medicine.
